# A Therapy System for Post-Traumatic Stress Disorder Using a Virtual Agent and Virtual Storytelling to Reconstruct Traumatic Memories

**DOI:** 10.1007/s10916-017-0771-y

**Published:** 2017-07-11

**Authors:** Myrthe L. Tielman, Mark A. Neerincx, Rafael Bidarra, Ben Kybartas, Willem-Paul Brinkman

**Affiliations:** 1 Delft University of Technology, Delft, Netherlands; 2 TNO Human Factors, Soesterberg, Netherlands

**Keywords:** E-health, Mental health, Virtual agent, Relational agent, Conversational agent, Virtual reality, Post-traumatic stress disorder, Therapy system, Ontology, Question system

## Abstract

Although post-traumatic stress disorder (PTSD) is well treatable, many people do not get the desired treatment due to barriers to care (such as stigma and cost). This paper presents a system that bridges this gap by enabling patients to follow therapy at home. A therapist is only involved remotely, to monitor progress and serve as a safety net. With this system, patients can recollect their memories in a digital diary and recreate them in a 3D WorldBuilder. Throughout the therapy, a virtual agent is present to inform and guide patients through the sessions, employing an ontology-based question module for recollecting traumatic memories to further elicit a detailed memory recollection. In a usability study with former PTSD patients (*n* = 4), these questions were found useful for memory recollection. Moreover, the usability of the whole system was rated positively. This system has the potential to be a valuable addition to the spectrum of PTSD treatments, offering a novel type of home therapy assisted by a virtual agent.

## Introduction

Post-Traumatic Stress Disorder (PTSD) is a mental disorder following one or more traumatic experiences. It is characterized by recurring intrusive memories, avoidance of reminders of the trauma and a persistent negative mood [1]. Cognitive Behavioral Therapy (CBT) with exposure is one of the most widely used treatments for PTSD. It relies on active recollection of the memory of the trauma to reduce the automatic fear response and facilitate cognitive restructuring [2–4]. Despite the existence of well-documented treatment, many PTSD patients don’t seek help. Stigma on mental health-care is high, especially amongst veterans [5], and issues such as travel times and cost can form further barriers to care. A stand-alone home-therapy system can therefore fill an important gap by providing a treatment which is easily accessible, privacy sensitive and cost-effective. Although many e-solutions for mental health are being developed, fully autonomous systems offering exposure therapy for PTSD are rare. The multi-model memory restructuring (3MR) system for home therapy for PTSD is one such system [6, 7]. In this paper we present 3MR version 2 (3MR_2), which incorporates several new elements such as a virtual agent and a question system for trauma recollection.

The original 3MR system was designed for in-clinic use, where the patient works together with a therapist in a face-to-face setting. Its goal was to support this therapy by facilitating trauma recollection and storytelling. The system was developed using expert input and included three main functionalities, namely a timeline, diary and 3D world editor. Memories could be added to the timeline to form an overview, while pictures, text and maps could be added to a diary, and a basic 3D version of the memory could be created in the 3D editor [6, 7]. The patient could work on these environments with the therapist, but also at home as part of homework assignments. By creating a visual representation the therapist would also get a better understanding of the patient’s experiences. This combination of exposure platforms was novel to 3MR, but several other technology-driven methods exist for exposure therapy. The most common is virtual reality (VR), which has had some very promising results [8, 9]. The main drawback of traditional VR therapy for PTSD is that the virtual environments are pre-created, and therefore difficult to match with personal memories. Although feasible for a specific group of veterans, most other patient groups generally do not share similar memories. In order to better personalize VR therapy, several platforms have been developed wherein therapists can build a virtual environment for their patients [10–12]. However, the original 3MR system shifts this task from therapist to patient, changing the main therapeutic component from the experience to the creation of a virtual world, which requires triggering the memory in an active way. As patients create their own 3D world, it facilitates the creation of a very personal autobiographic virtual environment. This shift also makes these environments suitable for home-use, as well as use in a stand-alone therapy where no therapist is present. The new 3MR_2 system expands on the concept of personal memory recreation by further developing both the diary and the 3D editor.

As 3MR_2 is designed for home-use instead of the original in-clinic use with a therapist, two additional main functionalities are necessary. Firstly, because the system is fully autonomous, it requires some form of procedure to ensure patient safety [13]. With the rise of technology in mental-health care, new ethics guidelines also need to be in place. Patient safety is a particular concern for systems that display some level of automation. Many systems have safety checks not in the system itself, but in the procedure surrounding its use. These checks can take the form of exclusion criteria, but also regular email or phone contact with a clinician [14–17]. In this way, it is still a human who provides the safety support. An alternative is to include all safety checks in the system itself without any human in-the-loop. These checks can take the form of questionnaires and crisis management options within the system [16]. A combination of these two solutions is to facilitate monitoring by a clinician through the system. In this situation, the system itself monitors the patient but a therapist uses the information gathered to ensure patient safety. An example of this situation is given by Robinson et al., where a clinician monitors distress scores in an application for students at risk for suicide [18]. A similar approach is taken by the 3MR_2 system; questionnaire scores can be monitored by clinicians, who make the call to interfere if patient safety is in question.

Secondly, a home-therapy system requires some form of interaction and guidance. The first goal of this interaction is to inform the patient and provide the rationale behind the assignments. The second goal is to assist the patient with memory recollection in a personalized way. Virtual agents have been gaining popularity in health applications as a way to add a social and personal aspect to systems. The addition of virtual agents has been shown to have a positive effect on attention [19], adherence [20, 21] and likability [22, 23] of applications. To assist with memory recollection during exposure, some knowledge of traumatic events is necessary. Ontologies provide a good way to add domain knowledge to computer systems [24, 25]. Through combining multiple-choice and open questions, knowledge can be gathered from the patient while still retaining a natural interaction between patient and system [26]. A combination of these paradigms has been shown to result in a question system which can elicit greater detail in the responses [27]. In the 3MR_2 system, a virtual agent is added to provide assistance and personalization, employing an ontology and a structured dialogue to pose the correct questions while assisting in memory recollection.

In the rest of this paper, we will present the 3MR_2 system in more detail. We will first expand on the monitoring function and the additions to the diary and virtual environment already present in the original 3MR. Secondly, we will describe the virtual agent and specifically the question system it employs to assist the patient during exposure. This paper will conclude with the results of a usability study of the system with former PTSD patients.

## 3MR_2 system

The 3MR_2 system offers therapy for PTSD. It includes the exposure environments introduced in the original 3MR, namely a timeline with memories which can be both described in a digital diary and re-created in a 3D WorldBuilder. The 3MR_2 system introduces monitoring within the system via questionnaires, improvements to the virtual environment design and a virtual agent to guide users through therapy and assist in memory recollection. It is specifically aimed at either victims of childhood sexual abuse (CSA) or war veterans. Differences consist of wording in some texts (e.g. ‘When you think back on your deployment’ vs. ‘When you think back on the period of the abuse’), the possible content of the virtual environment (e.g. models of tanks in the war version, children’s beds in the CSA version) and the concepts in the ontology on which the question system is based.

**Fig. 1:**
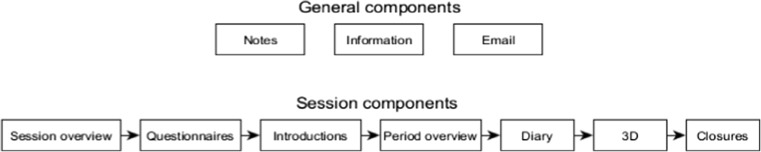
Outline of the components in the 3MR_2 system. In a typical session, the patient starts with the session overview page before answering several questionnaires. Afterwards, they receive an introduction to the session and goes to the period overview, showing a list of the added memories. In the diary, one memory can be described in detail, after which it is also recreated in the 3D WorldBuilder. After the memory is described and recreated, the session is closed. The notes, general information and e-mail pages can be accessed by the patient at any time during a session

Figure 1 shows an outline of the different components within 3MR_2. During a session, the patient is guided through the session components to perform the different therapy tasks. The general components can be accessed any time if the patient so wishes, but are not included in the therapy flow. These include general information on the system, a possibility to read back the psychoeducation and an e-mail function to contact the helpdesk or therapist with questions. The patient is not required or advised to use this e-mail function, but it is included to give the patient the option to reach out. The system always opens on the session overview screen, where patients can see a list of their sessions and their planned dates. Via this screen, they can start their next session. The patient always starts with the questionnaires. These are the post-traumatic stress disorder checklist (PCL) [28] and the patient-health questionnaire (PHQ) for depression [29]. These questionnaires are taken every session and visible for the therapist who monitors the patient. After the questionnaire, the patient is asked to read a brief introduction to the session explaining what the content is, and to select which feeling is predominant at the moment. Next, the patient is presented with the period overview, which lists the periods they have worked on in the diary. At the top of the screen these periods are also represented on a timeline. During the sessions where patients work on their memories, they can use this list to go to the appropriate diary page. In this diary page, the virtual agent asks questions to guide the patient through filling in the diary. After completing the diary, patients can use the WorldBuilder to recreate or review the 3D version of the memory they just described. This is the final exercise of the session. Patients then close the session by again selecting which feeling is predominant now, to illustrate changes between the beginning and end of the session. After that, they can return to the start page to close the program. Slight variations in this procedure exist, for instance the last session focuses on relapse prevention and does not actively include working on the memory. The full therapy consists of 12 sessions, starting with two sessions to get familiar with the system and memory recollection. The following eight sessions gradually introduce the exposure elements, working on three traumatic memories, which are increasing in impact. The final two sessions are aimed at review, reflection and a brief relapse-prevention. Exactly when and how the sessions are scheduled depends on the clinical setting that the system is applied in.

### Monitoring

**Fig. 2:**
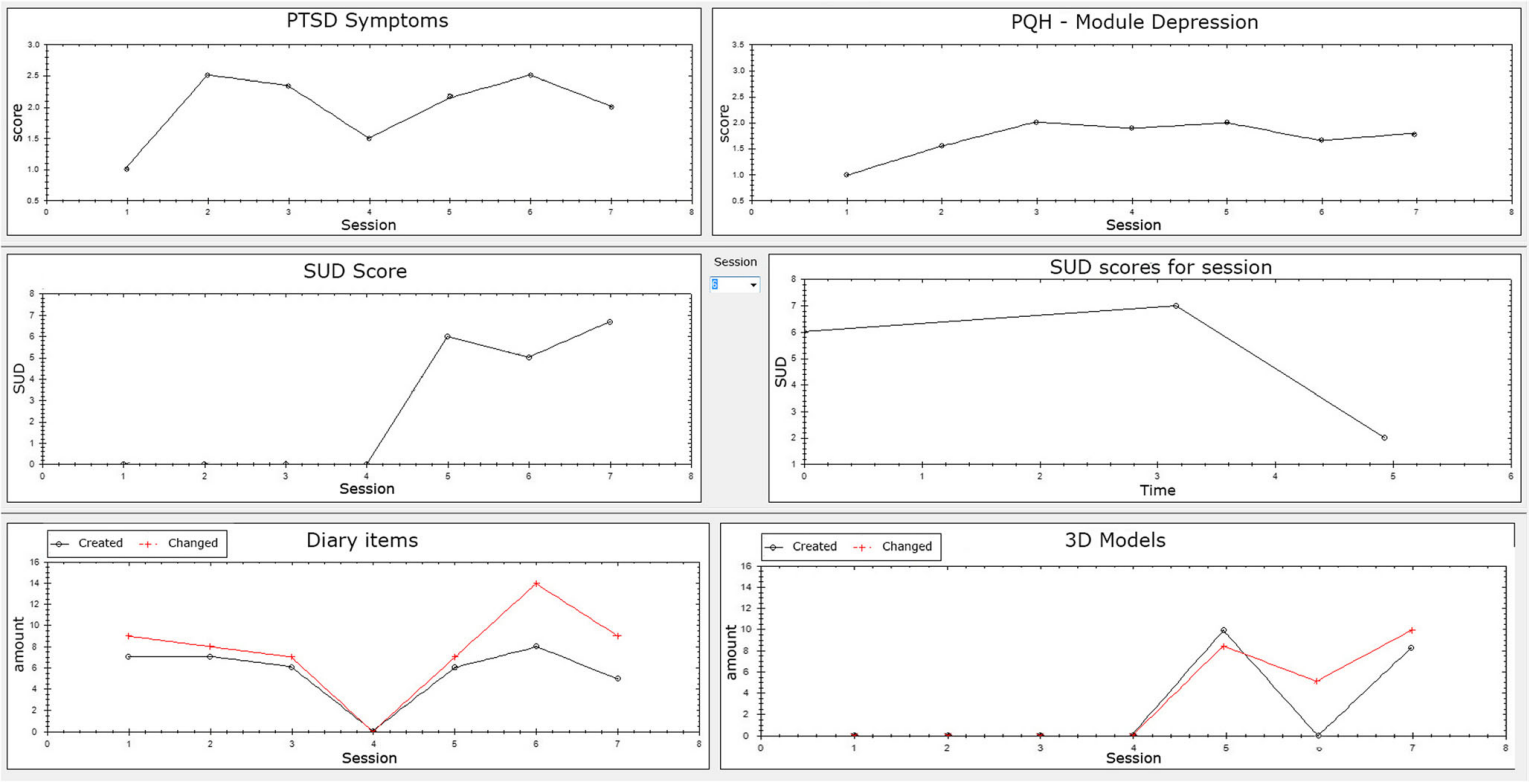
Screenshot therapist system showing example data for PTSD symptom scores, Depression symptom scores, SUD scores and activity data of diary

Although the 3MR_2 system is aimed at stand-alone home therapy, a clinician still has a monitoring role. Patients fill in the PHQ and PCL at the start of every session. Additionally, the system asks the patient to enter their subjective unit of discomfort (SUD) score [30] before, during and after each exposure session. Finally, the system collects activity data from the diary and 3D environment, including the number of items added or viewed during a session. A monitoring tool shows these scores in graphs, as shown in Fig. 2. If the therapist deems the scores serious enough to intervene they can do so. This can, for instance, be when the depression scores rise very high, or if the patient does not show activity in the diary when they should have performed a session. Additionally, the patient has the option to send a secure message to the therapist through the same server, if they would wish. The therapist views these messages with the same monitoring tool.

### Exposure environments

**Fig. 3:**
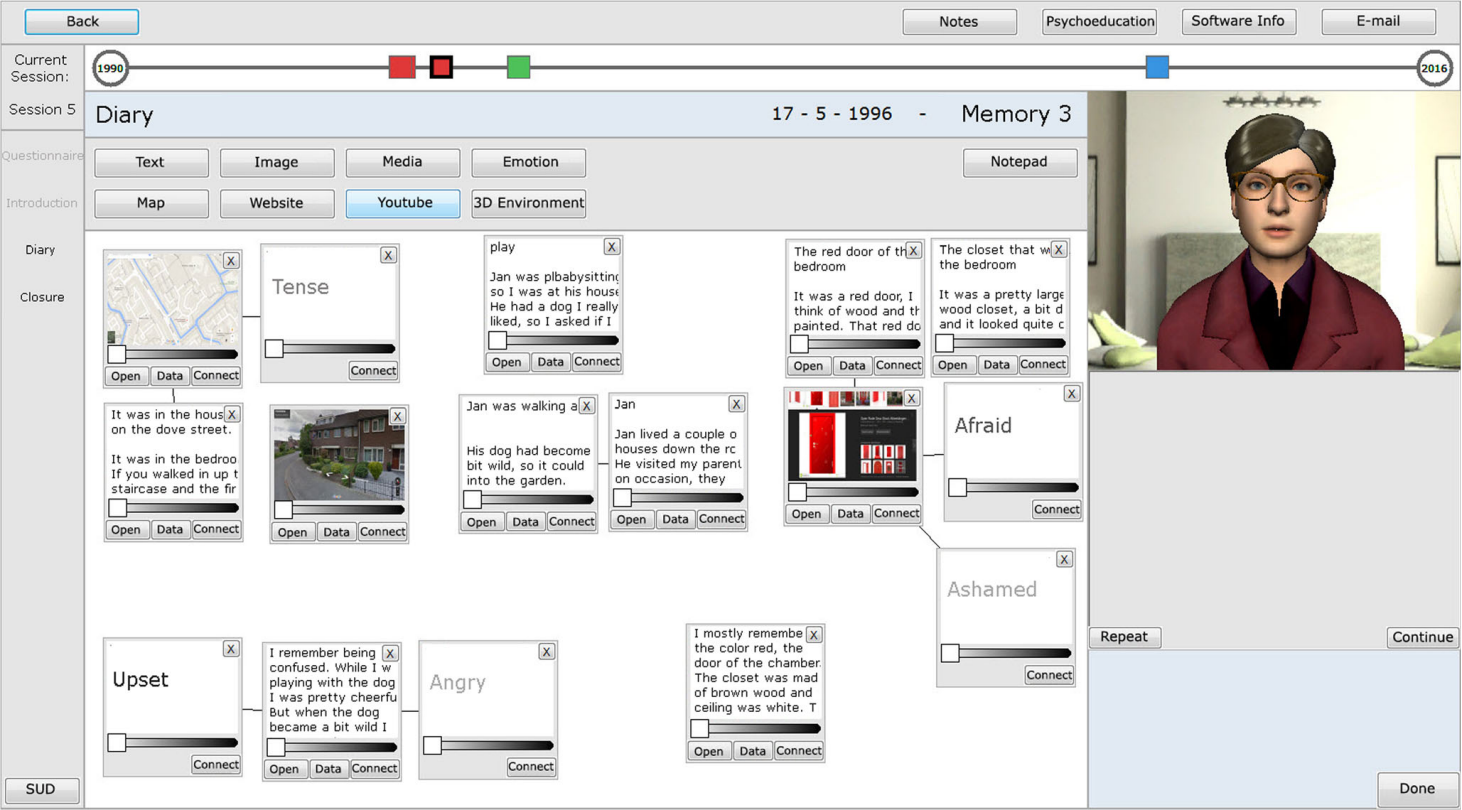
Diary, what it could look like when filled. Translated from the original Dutch version

The 3MR_2 system has two main exposure environments, a digital diary and a 3D tool, the WorldBuilder. Both are based on the environments in the original 3MR, but have been completely re-designed to improve usability, and add additional functionality to increase the possibilities within the environment. The digital diary can be filled with text, images, media, emotions and web-items such as maps and YouTube clips. The emotion items are a new addition compared to the original 3MR system and appear as words in the diary. This function was added because describing emotions and feelings is an important part of writing about memories [31]. Included in the emotion function is an option to relate the emotion to feelings during the memory or feelings in the present. As in the original 3MR system, all diary items can be added via a menu at the top and appear as a movable thumbnail on the screen, which can be enlarged to focus solely on that item. If items are too confrontational for permanent display they can be darkened. A new function has been added to allow connections to be made between items, indicating a relationship. The diary is used to describe the memory in detail. When working on the traumatic memories, the virtual agent guides patients through filling the diary with the ontology-based question system. If patients wish to add items after the questions they are always free to do so though. Figure 3 shows a diary filled with items.

The WorldBuilder is 3D tool in which patients can build a virtual environment, recreating their memories. Where the original 3MR only used a birds-eye view, two perspectives have been added to the new WorldBuilder. Using a collection of 3D–models, patients can make a top-down sketch of their recollections. In addition to this top-down edit view, patients can view the scene from a birds-eye perspective, which allows for zooming and viewing the scene from different angles. When building the scene, the patients can select 3D models from a menu on the right and drop them in the scene, rotating and moving them to put them in place. The interface is designed such that people with basic computer skills can use the tool. A brief instruction video is included as well, to further familiarize users with the interface. The CSA version is developed for indoor scenes, the scale of items reflecting the size of a large to smaller room. The version for veterans on the other hand is designed for outdoor scenes. Both versions have a selection of textures for the walls and ground, including both indoor textures such as wooden or carpet floors, and outside textures such as dirt and grass. Additionally, buildings are offered. The CSA version uses European-style buildings specifically, while the veteran version also includes a broad range of eastern-styled buildings. Similarly, the CSA version includes regular vehicles such as a car and bike, while the veteran version includes a range of army vehicles such as trucks and tanks. Regarding furniture, the CSA version is equipped with a range of objects to build bedrooms, studies and bathrooms, while the veteran version only includes basic furniture. The human models in both versions come in different ethnicities, both including a range of male, female and child models. The veteran version also includes a selection of Dutch army models. Finally, both versions are equipped with a range of general objects such as toys and books for the CSA version, and crates and roadblocks for the veteran version. Aside from some additions in the model library, the new 3MR_2 system has several other novel functions. These are the function to watch the environment through the eyes of the different people in-scene, and the option to create different scenes of the same memory. These scenes can be used to represent a timeline of events.

**Fig. 4:**
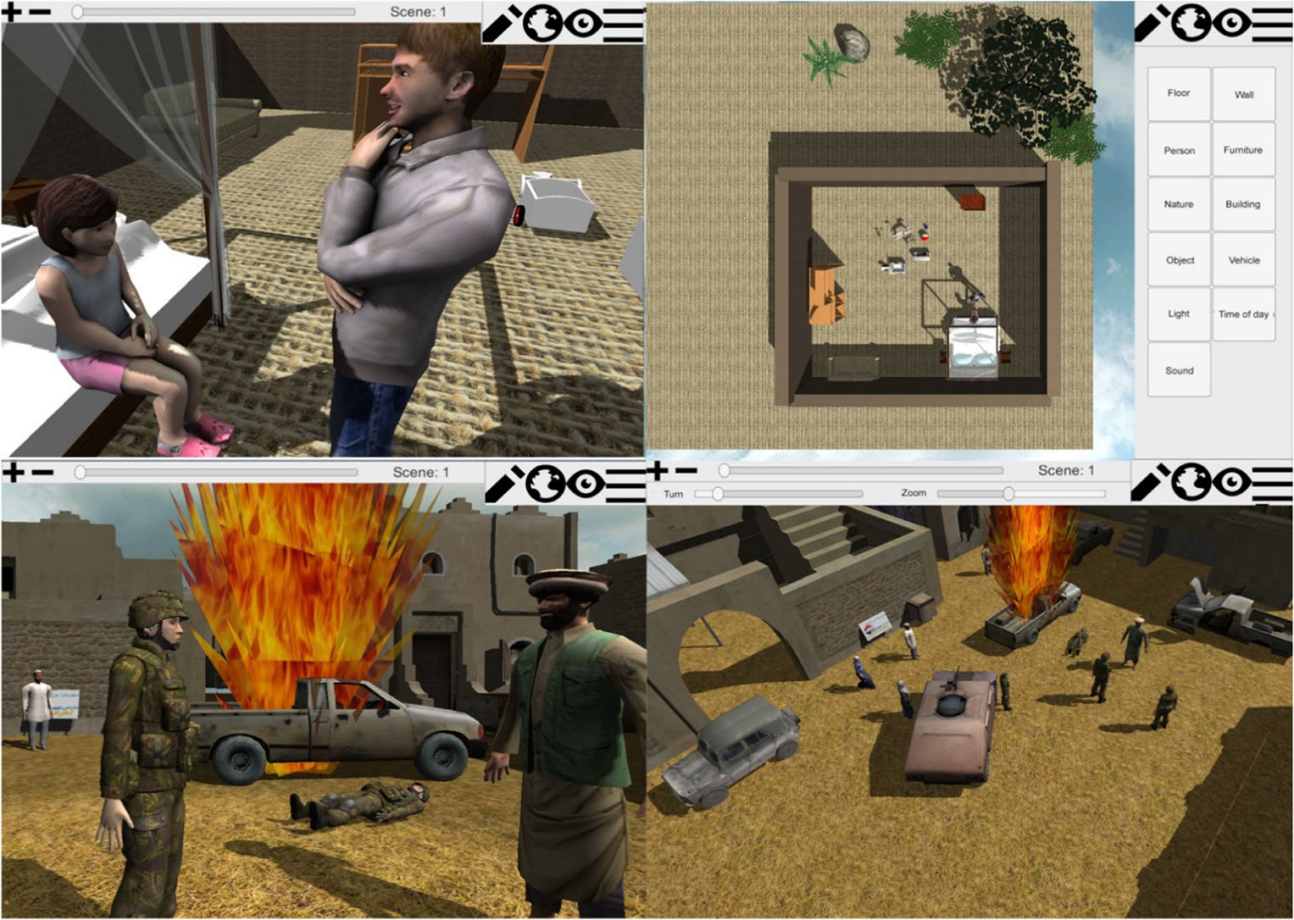
WorldBuilder. Top images are from the CSA version, in-scene person perspective (*left*) and edit (*right*). Bottom images are the war version. Left the in-scene Person perspective, right birds-eye perspective. Translated from the original Dutch version

**Fig. 5:**
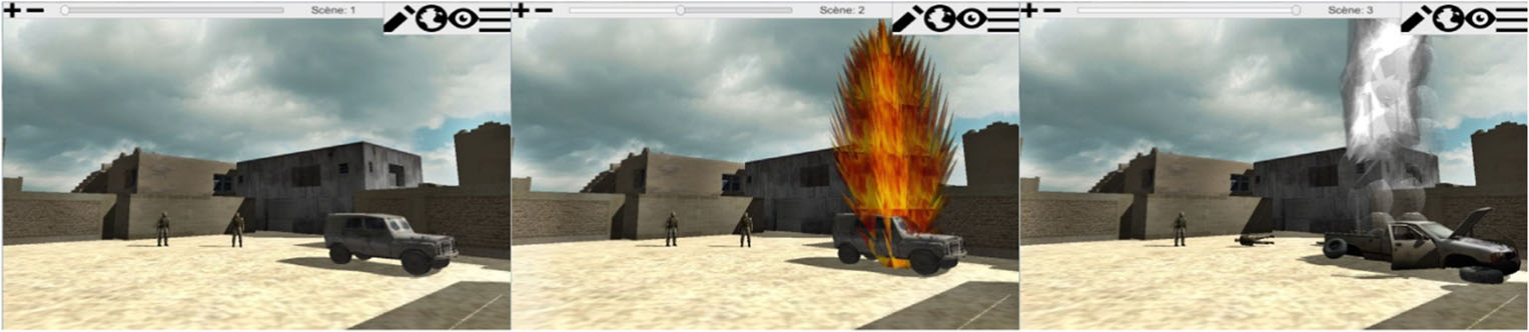
An example of three scenes telling a story of a jeep exploding in the WorldBuilder

Through changing the environment over scenes patients can tell their story. By clicking through the scenes, patients can watch the events play out in a very controlled manner. Patients work on one memory in two different sessions. In the first they are asked to create a static scene, placing floors and big objects first, small objects and people last. In the second session they are asked to review the memory and create the different scenes. Figure 4 shows various worlds created with the two WorldBuilder versions and the different perspectives. Figure 5 shows an example of a story told through three scenes.

### Virtual agent

**Fig. 6:**
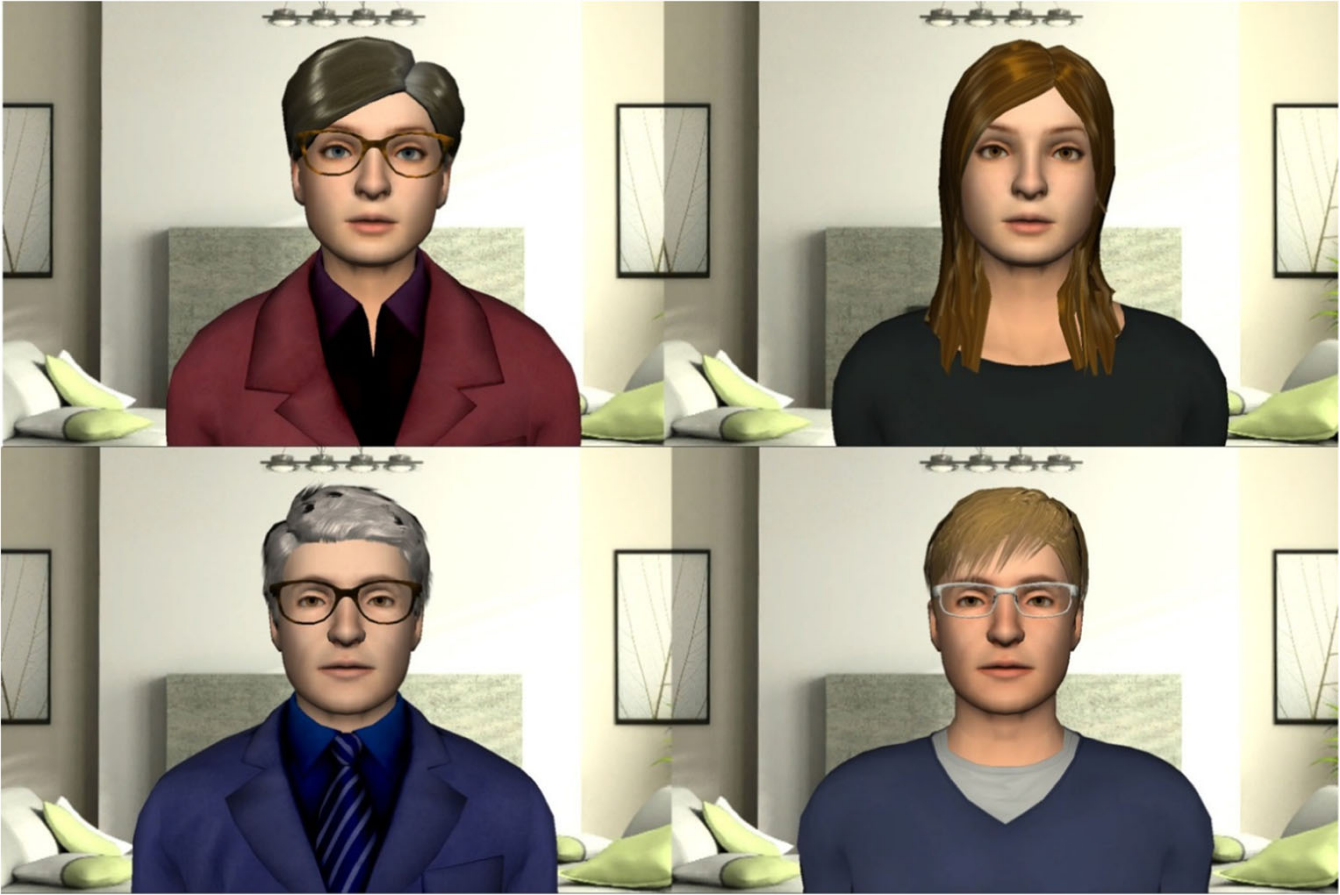
Possible virtual agents. Female abuse victims will be able to choose between the two female agents, male abuse victims between the two agents on the right. Male veterans are able to choose between the two male agents, female veterans between the agent on the top left and bottom right

At the beginning of the first session, patients pick their virtual agent. Four different agents exist, but all patients get a choice between two, depending on gender and patient group (Fig. 6). This pre-selection is made for several reasons, firstly so that the female CSA victims will not be confronted with a male agent, but also to enhance later satisfaction with the agent, as more choice may lead to lower satisfaction [32]. Aside from picking the gender and appearance, the patient also chooses a voice for the agent. The voices are generated by the Dutch text-to-speech system Fluency.^1^ All virtual agents display general idle behaviour and mouth movement while talking. The agent talks to the patient, but a repeat option exists which also displays the text. The user only responds with actions, or in the case of a multiple-choice question, with selecting a pre-set answer. The virtual agent has several key functions. First, it acts as a guide through the system and the therapy, telling the patient what to do and where to click. For instance, the virtual agent will ask the patient to fill in the questionnaires. Second, it provides background information on the therapy-concept, explaining why certain tasks need to be performed. For instance, when asking the patient some reflective questions after building the 3D environment, the agent also reminds people that they should not avoid thinking deeply about their memories. Third, it assists the patients during the exposure by asking personalized questions within the diary environment.

The virtual agent is not present while patients work on the 3D WorldBuilder, but does have a couple of functions related to this environment. First, if patients wish to take a break during the exposure they can select the option to have a relaxation exercise that is led by the virtual agent. Additionally, the virtual agent asks a number of questions after the 3D world has been finished. Several questions are general and focused on the experience, such as what the patient hears and feels when looking at their world. However, the virtual agent also notices some significant events in the 3D environment. It notices when objects move between scenes, when people models are laying down instead of standing, and when explosion models are added. On these occasions, it specifically asks the patient what this event meant for them.

The goal of exposure within PTSD therapy is that patients confront their memories and experience that thinking about these moments is possible. It is important that people think back in detail, so personalized and detailed questions are very helpful. To do this, the virtual agent employs an ontology-based question system. Past research has shown that such an ontology-based question system is able to elicit more detailed descriptions in memory recollection [27]. The ontology in the 3MR system represents knowledge about traumatic memories, differing slightly for either the veterans or CSA victims. Four different versions exist of the military ontology, one for Afghanistan, one for Bosnia, one for Libya and one not related to a specific deployment. These locations are chosen to best represent the Dutch missions.

**Fig. 7:**
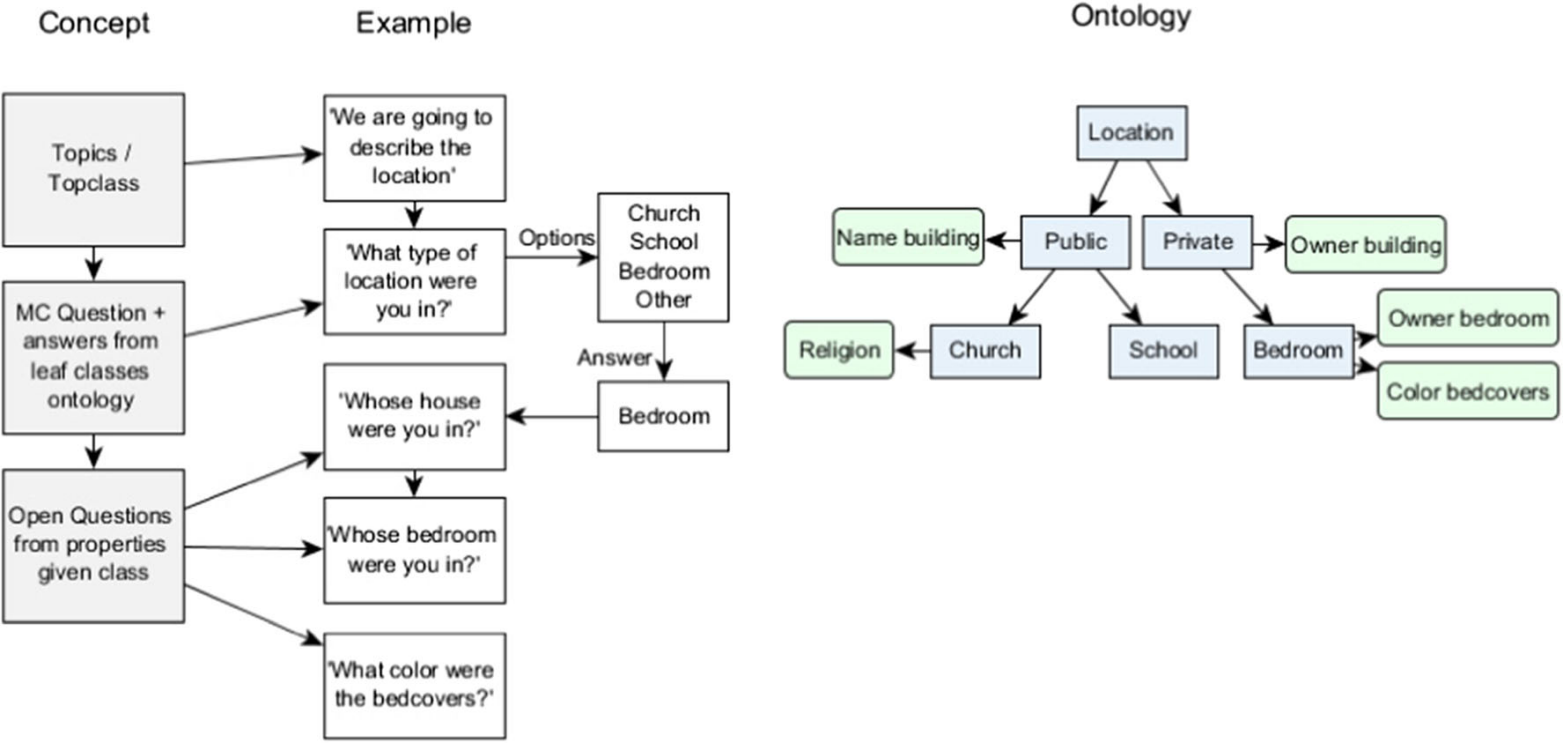
The process used within the question system. Based on the simplified ontology on the right, blue represent the classes (concepts) and green the properties of those classes. Given the topic of location, the first question will be multiple-choice, with as possible answers the leaf classes in the ontology. If the given answer would be ‘other’, the question is posed again with the options one level higher in the ontology (public/private in this case). Given the answer ‘Bedroom’, the following questions are open and correspond to the three properties of the Bedroom class

The ontology is based around the topics of location, objects, people, actions, senses and emotions. Each of these concepts is represented by a hierarchy of classes in the ontology. For example, the top-class location has subclasses for inside and outside locations. For the CSA version, the inside location has classes such as house, school and church, while the war version focuses more on outdoor locations and includes a road and base camp. The virtual agent introduces each topic with a multiple-choice question to determine which class is relevant. Using that answer, it asks open questions corresponding with each of the properties of this class, which the patient can answer in the diary. For instance, if the location is a school the agent will ask if the person also went to this school, and if they can remember places in the school where they often went. If it is a road, the question will ask where the road goes and if they were here often, etc. Figure 7 shows a schematic example of this process. This ontology-based question system is used to fill the diary for the trauma recollection, and guides the patient through this process in two sessions. In the first session, the virtual agent asks about location, objects and people. Although answers to the open questions are entered in the form of text, the agent will also ask to add maps or photos if these are available. In the second session, the agent will ask about what happened in the scene, what the patient smelled and heard, and finally what emotions they felt. Regarding what happened, the agent asks the patient to select verbs about what they themselves were doing, and what the others were doing and follow up from there. For instance, if the person would select ‘shooting’, the agent will ask what that person was shooting at, but also what happened right before and after this action. For emotions, the agent will ask how the person felt during the memory, but also to describe how they feel about it now and what has changed and why.

## Evaluation

Usability was evaluated in two stages. An initial usability test with healthy participants was performed for the virtual agent and diary environment. Based on this test, improvements were made to the system. An additional study was done with former PTSD patients studying both the usability of the system and its usefulness for recollecting traumatic memories.

### First usability test

Three healthy participants were recruited for an initial usability test, all were researchers or students at the Computer Science department, two had a background in psychology, one in computer science. They performed the first therapy session, in which a positive memory is described. This session did not yet include questions from the question module or the 3D WorldBuilder. No explicit instructions were given. Participants were asked to ‘think aloud’, and an experimenter was present to note all comments and what went wrong. Based on these usability tests, small improvements such as button placement were made to the system. Two instructional videos were made using the usability input, one describing the general system, and one specifically for the 3D WorldBuilder.

### Second usability test

A second usability test was performed with former PTSD patients. Its goal was threefold, firstly to study the general usability of the system and its components. The second goal was to study how much the system elements contributed to therapy according to users. The final goal was to study how useful and appropriate the questions generated by the question module were in recollecting traumatic memories. The design of this study was approved by the ethics committee of Delft University of Technology.

#### Participants

4 participants were recruited via practicing therapists. All participants had in the past followed therapy for PTSD. Participants 1 and 2 were war-veterans (both male), participants 3 and 4 had experienced childhood sexual abuse (both female).

#### Procedure

All participants first received general information on the 3MR_2 system, what a full therapy would look like and what was expected of them during the experiment. After this, participants followed the entire first therapy session, which is aimed at familiarizing oneself with the system by describing a positive memory. Following the first session, participants skipped ahead and followed parts of two sessions in which one traumatic memory is described. During these sessions, participants were asked to keep one personal memory in mind. They were requested to answer all multiple-choice questions, but none of the open questions. These were only rated in terms of usefulness. The experiment ended with a general questionnaire.

#### Measures

The first measure was how useful the questions generated by the system were for remembering the trauma. All questions were rated on an analog scale ranging from *works against recollection* to *helps a lot recollecting*. The center point was marked as *question has no effect.* All questions were rated immediately after asking. The second measure was how useful and understandable the functions in the program were. Two questions were posed on an analog scale, firstly asking how useful the function was from *detrimental* to *helps a lot*. The second asked how understandable the function was, ranging from *confused me* to *very understandable*. The final questionnaire was the Dutch version System Usability Scale [33], applied to the whole 3MR program, answered on a 5 pt. Likert scale.

#### Analysis

All statistical analyses were carried out with R version 3.3. Before the analysis, the usefulness scores for the questions were transformed to range from −50 to 50, with 0 as the neutral point so deviation from 0 could be tested. Multilevel analyses taking participant as a random intercept were conducted on the usefulness scores for the questions, the usefulness scores of the system components and the usability scores of the system components. The analysis of the usefulness of the questions only included a fixed intercept. In the analysis on the usefulness and usability of the system, system component was included as a fixed effect to study if this factor influenced the result.

#### Results & discussion

**Fig. 8:**
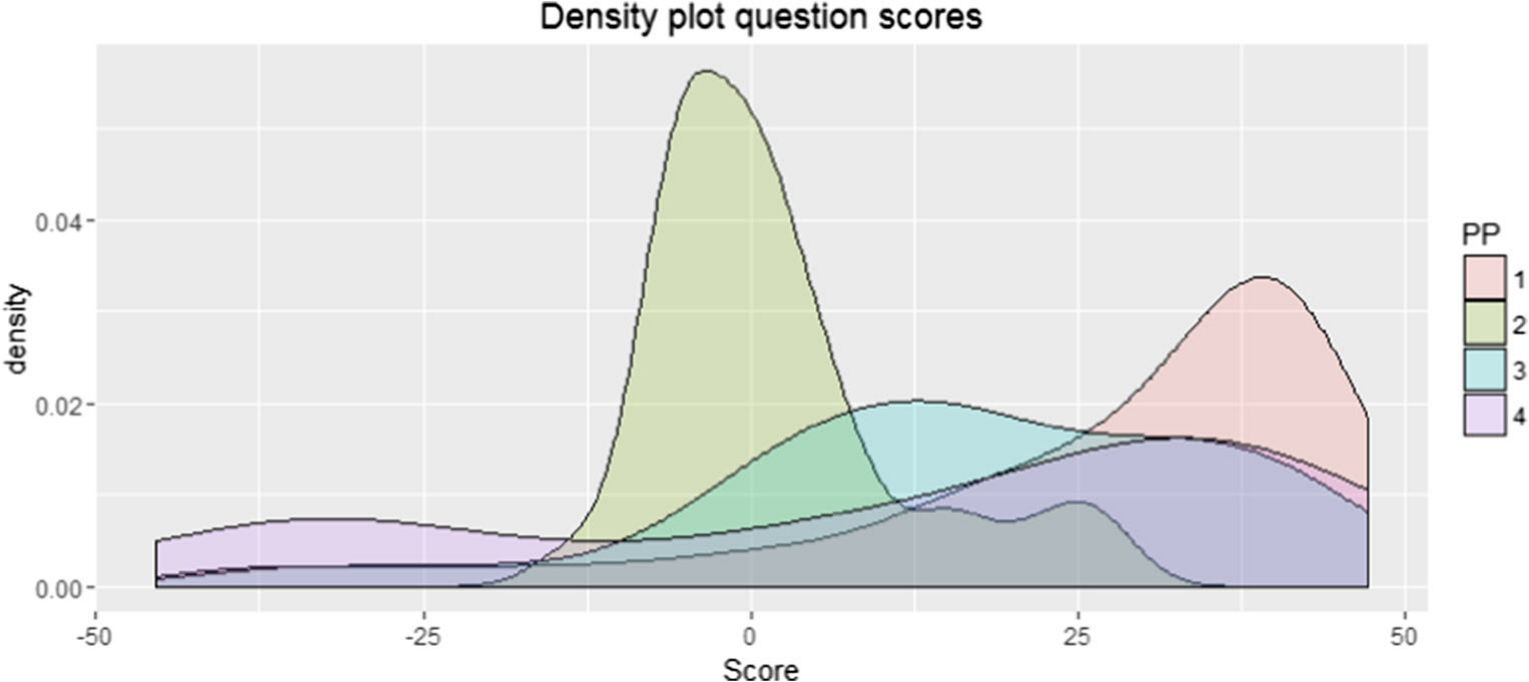
Density plot of the scores given to the questions posed by the question system. Participants 1, 3 and 4 scored the majority of questions above 0, but a couple got quite low scores. Participant 2 scored nearly all questions around 0, a couple slightly higher but none very low

The analysis of the usefulness ratings of the questions revealed that on average, participants found the questions helped them to recall their memory (*Mean* = 15.11, *SD* = 22.93, *F(*1140) = 10.03, *p* = 0.002). A significant variation between participants was found, however (*SD*_random intercept_ = 8.37, 95% CI [3.72, 18.83]). Figure 8 shows a density plot of the given scores for each participant, showing that participant 1,3 and 4 have a relatively similar pattern. Participant 2, however, gave nearly every question a score surrounding 0, with none below −25 and a few above 25. The questions receiving low scores were individually reconsidered and revised.

The analysis of the usefulness scores revealed that on average, participants found the system useful (*Mean* = 16.32, *SD* = 16.17, *F*(1,20) = 13.98, *p* = 0.001). No difference was found between the different system components (*F*(5,15) = 1.34, *p* = 0.30). However, ratings varied significantly between participants (*SD*_random intercept_ = 6.12, 95% CI [1.54, 24.36]). A closer inspection showed that only participant 2 found functions to be detrimental to the therapy, namely the virtual agent and the instruction video. This might partly be explained by the fact that he had worked with a system without either virtual agent or question module in the past, and was therefore used to working with the diary alone. This might have resulted in impatience with the virtual agent and questions. Nevertheless, this preference for a system with less guidance might also exist in people without prior experience with 3MR.

The usability scores reveal a similar pattern to the usefulness scores. On average, the system was rated as well usable (*Mean* = 21.98, *SD* = 20.71, *F*(1,20) = 7.26, *p*. = 0.014). No significant difference was found between system components (*F*(5,15) = 0.61, *p* = 0.69), while participants did significantly vary in their ratings (*SD*_random intercept_ = 14.96, 95% CI [6.78, 32.99]). The rating on the System Usability Scale ranged between 73 and 75 for participants 1, 2 and 3, which can be labeled as above average. The exception was participant 3 who gave a rating of 55. This is probably caused by a bug only this participant experienced in the 3D WorldBuilder (the scale of the 3D models was wrong).

## Conclusion & discussion

In this paper we described the 3MR_2 system, a therapy system for PTSD patients. The system contains two exposure environments, a digital diary and a 3D WorldBuilder in which memories can be recreated. During a 12-session therapy, a virtual agent guides and assists patients with their therapy tasks, employing an ontology-based question module. A human therapist is involved only to monitor progress.

Initial evaluations revealed that the system was usable by both non-patients and former PTSD patients. These evaluations did reveal small usability concerns, which were then resolved. They also exposed some differences in personal preferences, one participant strongly preferred working at his own pace without much guidance. The current system is less appropriate for patients with such a working style, which should be kept in mind for future use. The questions generated by the question module were deemed useful. The evaluations presented in this paper were only concerned with usability and the appropriateness of the generated questions, but did not look into therapeutic effectiveness. A benchmark study is currently being set-up to test if the 3MR_2 system is successful in significantly reducing PTSD symptoms.

Although the 3MR_2 system is specifically designed to treat PTSD, components are also relevant for other domains. Firstly, providing safety and reducing human resources are very important in e-mental-health [34–36]. The 3MR_2 gives an example of limited human monitoring, which achieves both goals. Secondly, the exposure environments present in 3MR_2 provide a novel view on technology-assisted exposure. Many studies have been done with VR environments for exposure therapy [9], but the concept of patients themselves recreating these worlds is novel, and might solve the difficulty of building worlds relevant to different patients [8]. Although embedded in a home-therapy in 3MR_2, both the diary and the 3D WorldBuilder could also be used as tools in regular therapy. Thirdly, the 3MR_2 system incorporates a virtual agent that can pose questions aimed at memory recollection. Although exposure to memories is specific to PTSD therapy, other health applications for this technology do exist. One example is expressive writing, a therapeutic tool aimed at writing about negative memories [37, 31]. Another possible application might be found in the field of life review therapy, wherein reminiscence is used to alleviate mental health symptoms, for instance in older adults with depression [38].

The 3MR_2 system provides opportunities to reduce the barriers to care. However, it is not suitable for all PTSD patients. Depression is a common comorbid disorder to PTSD, and suicide rates among PTSD patients are high [39]. Because patients work alone with the 3MR_2 system, it might be less suitable for patients with a history of suicidality. Similar concerns arise for patients which have substance problems [1], or which severely dissociate [40]. Another limitation to the 3MR_2 system is that patients cannot follow therapy while ignoring the virtual agent. Some people prefer more guidance than others, and some might not respond well to following a set course. Where a therapist might be able to steer the patient back on track, this is more challenging for a virtual agent.

Despite these challenges, we believe that the 3MR_2 system is a valuable addition to the spectrum of PTSD treatments. Through the use of new technologies, it offers a novel type of therapy which is convenient to patients and costs very little in therapist resources. Given the societal impact of PTSD, it may have a great positive effect on society.

## Footnotes

1
www.fluency.nl
